# Aligning spatiotemporal supply and demand of nature’s contribution to people (NCPs) for sustainable resource management

**DOI:** 10.1038/s41598-025-17652-4

**Published:** 2025-09-11

**Authors:** Matteo Riva, Felix Kienast, Adrienne Grêt-Regamey

**Affiliations:** 1 Planning of Landscape and Urban Systems, Swiss Federal Institute of Technology (ETH), Stefano- Franscini-Platz 5, 8093 Zurich, Switzerland; 2 Swiss Federal Institute for Forest, Snow and Landscape Research (WSL), Zürcherstrasse 111, 8903 Birmensdorf, Switzerland

**Keywords:** Ecosystem services, Flows, Resilience, Urban-rural interaction, Human-nature interactions, Ecosystem services, Environmental impact, Environmental impact, Sustainability

## Abstract

Sustainable resource management requires a dynamic understanding of how ecosystems meet human needs over space and time. This study presents an integrative approach that combines spatial and temporal analyses of Nature’s Contributions to People (NCPs), aiming to systematically assess the evolving alignment between their long-term supply and societal demand. By combining historical trajectories with current NCPs budgets and employing a clustering approach, we uncover emerging imbalances, shifting dependencies, and shared pathways within and between municipalities and larger regions. Our findings show that NCPs demand often outpaces supply, leading to increasing deficits and ecosystem stress, particularly in urbanized regions. Conversely, rural municipalities generally exhibit surpluses, though these are subject to shifting pressures over time. The observed patterns emphasize the importance of polycentric governance and proactive management strategies that account for both local sustainability and broader interdependencies. This approach provides valuable insights for policymakers and planners seeking to balance ecological integrity with societal needs in a rapidly changing landscape.

**Supplementary Information:**

The online version contains supplementary material available at 10.1038/s41598-025-17652-4.

## Introduction

Human well-being relies upon the different contributions provided by ecosystems, ranging from tangible goods such as food and water to services like climate regulation and recreational opportunities^1,2^. However, growing pressures from urbanization, population growth, changes in land use, and climate change increasingly limit these contributions^3^. This leads to growing spatial and temporal mismatches between the supply of natural resources, the capacity of ecosystems to provide a range of benefits, and the associated societal demands and expectations for these goods and services^4,5^. Urban centers, for example, often rely on distant rural areas to meet their demand for food, water, recreational space, and other services, creating telecoupled relationships that complicate resource management^6^. Effectively addressing these imbalances requires a comprehensive understanding of how human-nature interactions are shaped, maintained, and managed across different spatial and temporal scales.

Various analytical frameworks have emerged, including social-ecological systems theory^7, ^ecosystem services assessments^8^and urban metabolism^9^each providing unique insights into the dynamics between ecosystems and societies. While acknowledging the perspectives offered by other frameworks, in this study, we focused on the Nature’s Contributions to People (NCPs) framework^10–12^ to effectively quantify, visualize, and interpret the interactions between ecosystem supply and societal demand. The various ways nature contributes to human well-being are categorized in 18 NCPs, divided into three partially overlapping groups: regulating, material, and non-material NCPs^10–12^. When demand for NCPs exceeds supply, ecosystems can become overexploited, leading to degradation and a reduction in their capacity to perform essential functions over time. Conversely, when NCPs supply exceeds demand, the ecosystem is in a stable state and can absorb changes in demand. Assessments that map and quantify the resulting NCPs dynamics, from local to interregional scale, are increasingly used to capture these processes^13–16^ and inform management practices^17^.

Supply and demand of NCPs are not static; rather, they evolve and change over time. Temporal analyses thus serve as a valuable complement to spatial assessments, offering critical insights into the dynamic fluctuations of ecosystem services and the corresponding human demand for these benefits^10,18,19^. Considering temporal NCPs trends is also useful for understanding lag effects, whereby the consequences of ecosystem degradation, frequently initiated by overexploitation, may not immediately diminish supply but manifest gradually over time^20^. Yet, while numerous studies have assessed the supply and demand of NCPs, often combining them into budgets or indices^21–24, ^only few also incorporated temporal dynamics. Many of these assessments often focus exclusively on changes in NCPs supply^25^ or on temporal trade-offs between supply and demand for individual NCPs^26,27, ^while more recent studies combine both supply and demand values across multiple NCPs over time^10,19,28^. Parallel to these developments, clustering approaches, bundles and archetype analyses have increasingly been used to generalize socio-ecological patterns and support landscape-level planning by detecting groups of co-occurring features or socio-environmental conditions^29–33^. However, such studies have primarily focused on the spatial dimension, with less attention paid to temporal patterns^34^. When temporal information is included, this is often limited to a twofold comparison between past and present or focused solely on NCPs supply^32,34^. In contrast, the combination of spatiotemporal information of NCPs supply and demand with clustering techniques remains mostly unexplored.

**Fig. 1:**
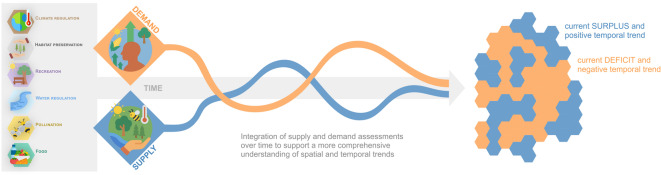
Conceptual overview of the study approach to support an integrated understanding of spatiotemporal trends in Nature’s Contributions to People (NCPs).

This study thus integrates spatial and temporal analyses of multiple NCPs to generate a more dynamic understanding of how NCPs supply and demand align over time and across space (Fig. 1). This combination of spatial and temporal information with clustering approaches allows to identify tendencies and emerging imbalances potentially leading to deficits, mismatches, resource depletion, or social challenges^3,4,35–40^. The integrative approach allows to uncover critical sustainability gaps and inform effective governance strategies^41^. Such insights are crucial because managing NCPs supply and demand interactions requires tailored solutions and coordinated efforts across multiple governance scales and sectors, a perspective known as polycentric governance^42,43^. Therefore, our integrative analysis aims to support such multi-level governance frameworks, offering practical insights for planning and managing sustainability challenges at municipal, regional, and potentially broader scales.

To illustrate the applicability of the methodological framework, we focused on the canton of Zurich (Switzerland), examining four decades of data to identify spatiotemporal trends in NCPs supply and demand. This region encompasses diverse landscapes, including urban centers such as the city of Zurich, peri-urban areas, and rural municipalities. The contrast between municipalities that have undergone rapid urbanization and land-use changes over past decades and those that have remained relatively stable provides a distinctive setting to analyze the interaction between NCPs supply and demand across different spatial and temporal scales. Finally, we discuss the broader implications of our findings for sustainable management and governance, emphasizing the necessity for polycentric governance strategies to effectively address the complexity and dynamic nature of human-nature interactions.

## Materials and methods

**Fig. 2:**
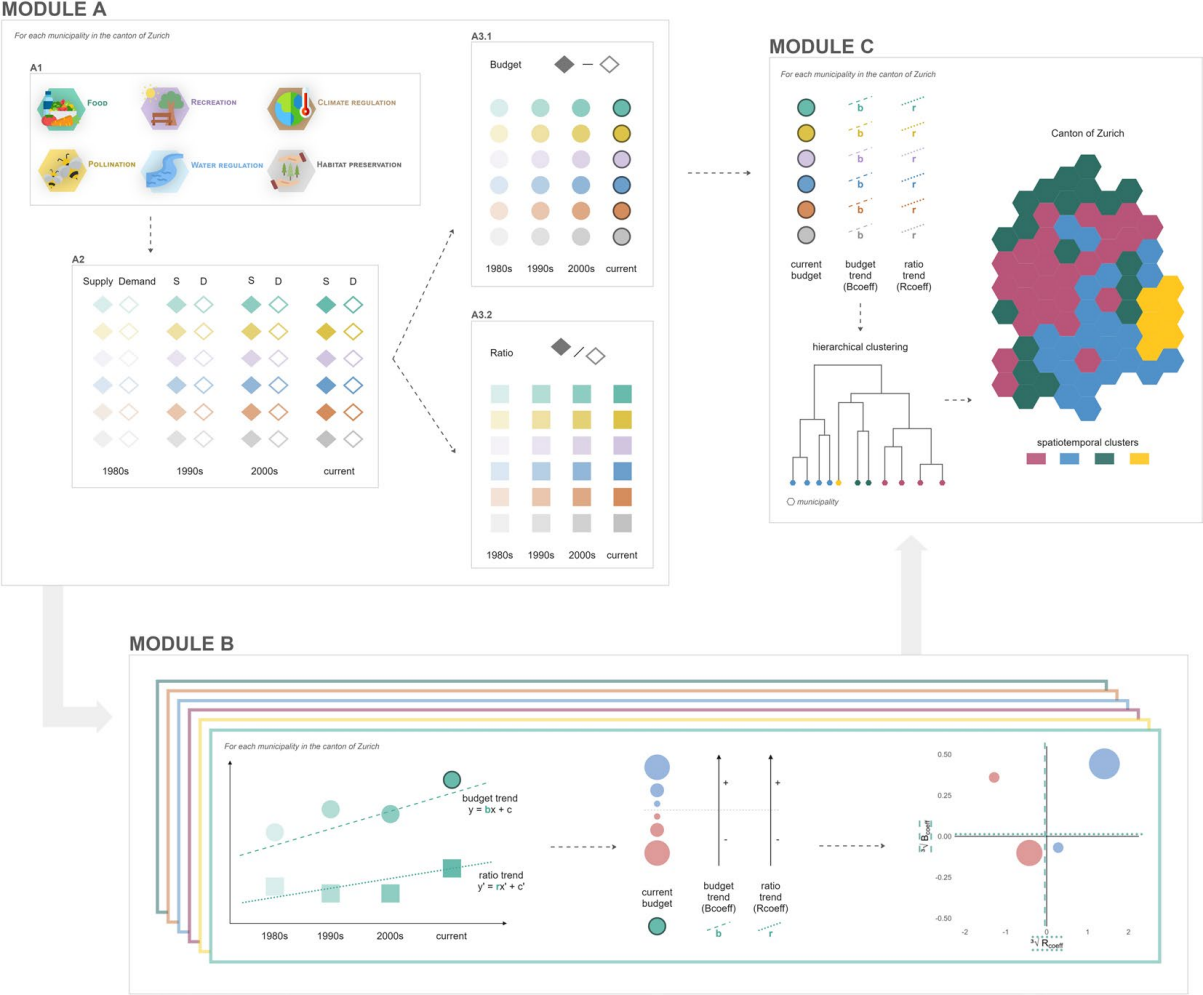
Overview of the study methodology. The abbreviations are cross-referenced with the related figures in other sections.

By synthesizing spatial and temporal information, this study provides a nuanced integration of NCPs supply and demand dynamics. Figure 2 provides an overview of the methodological steps, starting with the systematic identification of relevant NCPs based on regional policy documents (module A1), spatiotemporal quantification of supply and demand (module A2), combination into budget and ratio values (module A3), trend analysis (module B), and finally hierarchical clustering to identify municipalities sharing similar spatiotemporal NCPs patterns (module C). To improve understanding, the abbreviations for the various methodological steps are also referenced in each related figure in the results section.

### Selection of NCPs

**Fig. 3:**
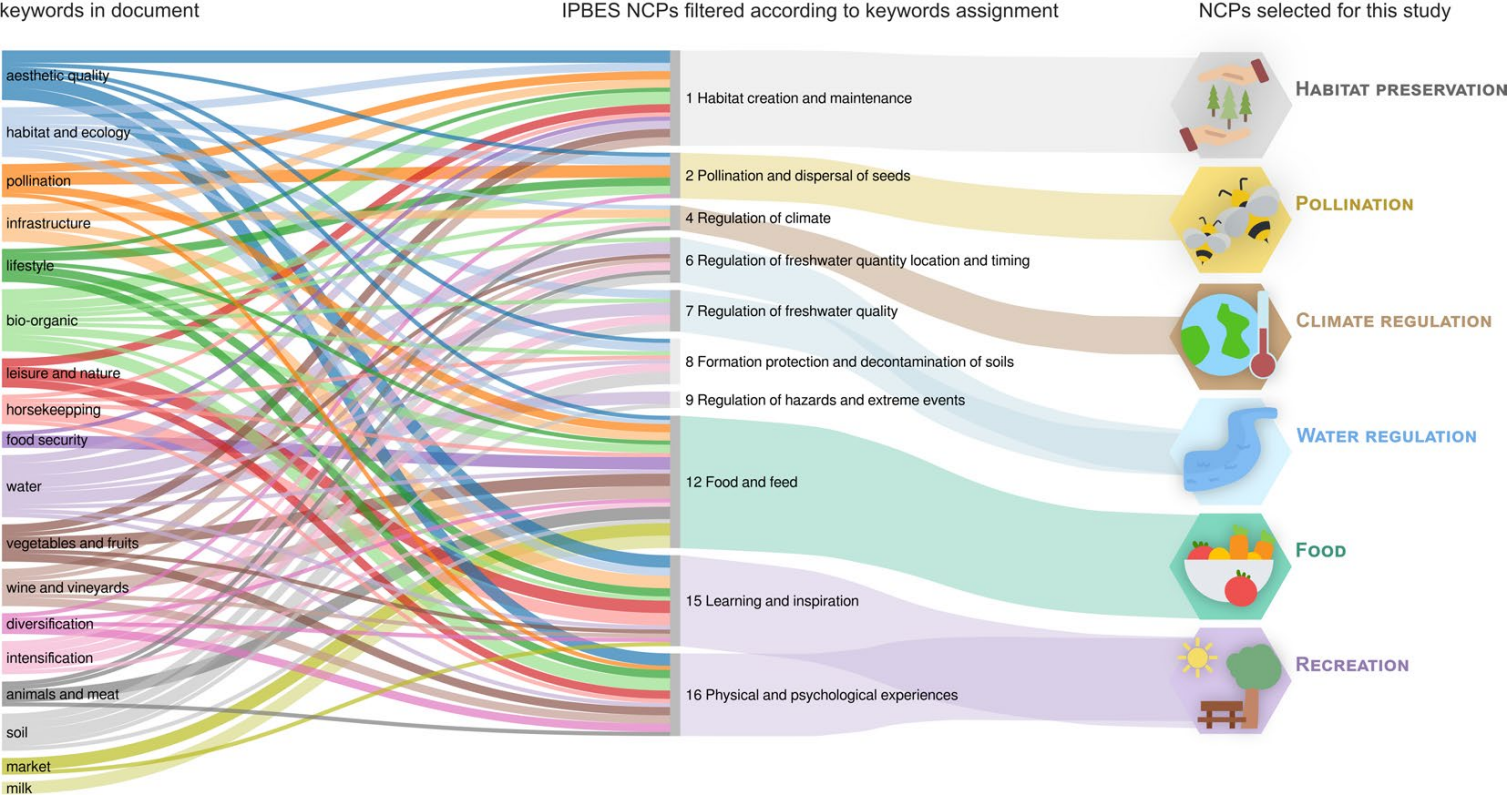
Process of NCPs selection from keywords identified in planning documents and assigned to related NCPs, to an initial filtered list of IPBES NCPs, followed by a final selection based on technical feasibility and data availability. In the methodological overview (Fig. 2) this step corresponds to module A1.

To define the scope of this study and focus on the relevant human-nature interactions, we conducted a qualitative content analysis of spatial planning documents^44–47^ (Fig. 2 module A1, and Fig. 3). This approach helped us to identify key environmental concerns, guiding the selection of the most relevant NCPs for the case study of the canton of Zurich. The implemented methodology was based on previous studies similarly assessing spatial planning documents^44–47^. The analysis began with the selection of relevant planning documents (as detailed in Supplementary material A), from which excerpts containing goals, directives, or expected trends were identified. Then, the *Langfristige Raumentwicklungsstrategie Kanton Zürich*^48^ was used to define a set of keywords associated with landscape, environmental and spatial planning, which guided the subsequent content analysis. For all identified documents, excerpts related to human-nature interactions were identified and coded according to these keywords. Each identified excerpt was then linked to the NCPs list from IPBES. Finally, the number of links per NCP was computed to obtain a rough estimate of the importance of each NCP in the identified planning documents. These insights, along with an evaluation of technical feasibility and data availability, determined the final selection of NCPs for this study (Fig. 3).

### Spatiotemporal NCPs analysis

In this study, we first examined four key components to evaluate the spatiotemporal trends of different NCPs.

- The** supply** and **demand **of each NCP (Fig. 2 module A2) represent the available provision and societal need for that beneficial contribution (for a detailed description see Table 1). To estimate these components, we used various models and datasets derived from the same source across the assessed years. Specifically, our study focuses on four timesteps: 1980s, 1990s, 2000s, and current. These timesteps match with one key dataset used in most of the assessed NCPs supply values, namely the Swiss Land Use Statistics data^49^. This dataset provides detailed and accurate information on the state and evolution of land use and land cover in Switzerland, with geodata available at the hectare level for the years 1979/85, 1992/97, 2004/09, and 2013/18 (released in 2021). The 2020/25 version is currently under development and is thus not included in this study. When continuous yearly values were available, these were averaged over the specific time windows mentioned above; otherwise, the best possible combination of available yearly values or single-year data points were used. By integrating this dataset with other sources and selecting information with similar time intervals, we ensured consistency in our spatiotemporal analyses, allowing for a robust evaluation of NCPs dynamics over time. Final outcomes were computed at the municipal scale for all four timesteps.
- The **budget** ($\:supply-demand,\:index\:[-1;1]$) is defined as the difference between the NCPs supply and demand, indicating the net balance within each municipality (Fig. 2 module A3.1). A positive budget reveals a surplus of supply over demand, while a negative budget highlights a deficit. To compute this, we subtracted the estimated demand from the supply at each time step, providing insights into whether NCPs provision is sufficient to meet societal needs over time.
- The **ratio** ($\:supply/demand,\:index\:[-1;\infty\:]$) is calculated by dividing the NCPs supply by the demand, offering a comparative measure, a percentage of the demand being met by the available supply (Fig. 2 module A3.1). While the budget provides absolute values in NCPs balance, the ratio highlights the efficiency of supply in meeting demand. For example, a decreasing ratio over time – even with a stable or increasing budget – indicates that supply is not keeping pace with rising demand.

**Table 1:** Overview of NCPs information and calculation approaches for supply and demand values. Each component was calculated for each timestep (1980s, 1990s, 2000s, current). The listed references refer to the methodological approach and the datasets used. In the methodological overview (Fig. 2) these components correspond to module A2.

NCP (code)	Component and indicator	Method	Unit	References
Pollination (POL)	*Supply* Pollinator abundance	Using the InVEST Crop Pollination model, compute the *pollinator abundance* and then calculate average value per municipality.	index [0; 1]	*Datasets* ^49–53^ *Methodology* ^17,27,54–58^
	*Demand* Crops dependence	Crops are divided into categories according to their theoretical need for pollinators. Calculate and rescale the percentage of affected crops in each municipality according to yield reduction estimates per crop category.	index [0; 1]	
Habitat preservation (HAB)	*Supply* Protected areas	Existing protected areas divided by level of protection (adapted from IUCN). Assign protection value to each category and compute average value per municipality.	index [0; 1]	*Datasets* ^23,49,59–67^ *Methodology* ^22,68–74^
	*Demand* Threats	Using the InVEST Habitat Quality model, compute the *habitat degradation*, rescale, and calculate average value per municipality.	index [0; 1]	
Recreation (REC)	*Supply* Recreation areas	Generate layers for different recreation features and average to obtain recreation value. Filter only values above literature-based threshold. Calculate available recreation space by multiplying value by the pixel area and a default reduction value (average usable space). Finally, average recreation values for each municipality.	m^2^	*Datasets* ^49,59,62,64–66,75–79^ *Methodology* ^4,19,22,26,36,58,80,86^
	*Demand* Recreation need/share	Use population density datasets to calculate total population in a buffer (representing potential recreation area in walk-distance) around each cell. Then calculate the total population need for recreation spaces and compute average value per municipality.	m^2^	
Food (FOD)	*Supply* Food production	For non-meat products calculate average yield and multiply by dedicated agricultural area. For meat products calculate average available meat per animal and multiply by number of animals per farm. Obtain values per municipality per product category and finally calculate the average food production per municipality.	kg	*Datasets* ^77,78,87–90^ *Methodology* ^18,19,21,81,91–93^
	*Demand* Food consumption	Gather average consumption per person and multiply by population data. Then calculate average value per municipality.	kg	
Water regulation (WAT)	*Supply* Water yield	Using the InVEST Water Yield model, compute the *water yield* (in mm/pixel). Calculate average value per municipality and convert to m^3^.	m^3^	*Datasets* ^49,94–98^ *Methodology* ^19,36,72,81,99–105^
	*Demand* Water consumption	Gather data on water demand per sector for each timestep. For household consumption link to population density maps. For all other consumption, compute per pixel average. Sum all water consumption in municipality.	m^3^	
Climate regulation (CLI)	*Supply* Net primary productivity	Use Net Primary Productivity (NPP) as proxy for carbon sequestration. NPP in tons carbon extracted from MODIS Terra satellite images (MOD17A3HGF). Before 2000 no such satellite image is available: linear regression was used to estimate NPP for previous timesteps and to ensure consistency.	ton	*Datasets* ^77,106–109^ *Methodology* ^19,22,81,85,93,102,110–113^
	*Demand* Carbon emissions	GHG emissions gathered from municipal data for current timestep and Climate Watch for past years. Computation of CO2equivalent per capita for each municipality and conversion to carbon.	ton	

To improve clarity and understanding, supply and demand results for some NCPs (food, water regulation, climate regulation) were calculated as per capita values, before determining budget and ratio indexes. This transformation mitigated the disproportionately high absolute values observed in Zurich and Winterthur, which would have otherwise dominated the dataset and overlooked patterns in less populated municipalities. In addition to this technical reasoning, normalizing NCPs related to consumption by population is widely adopted in ecosystem services research to relate environmental benefits to beneficiaries^21,114,115^. This additional step enables a fairer comparison of provision and use across municipalities, illustrating how NCPs address the needs of residents in various contexts.

To assess and evaluate these four components (supply, demand, budget, ratio), it is essential to consider the interactions between supply and demand, which result in spatial flows connecting provisioning areas with beneficiaries. These flows are commonly divided into categories, such as commodity transfers, biophysical flows, information network, or direct access, each characterized by specific attributes related to discrete processes in space^13,16,38^. These flow types assist the choice of indicators to calculate and represent supply and demand, as each type reflects different processes and interactions between ecosystems and people. For example, biophysical flows may be best captured through ecological indicators, while commodity or access-based flows might require social or infrastructure related data. Further, as described by Schirpke et al.^4^ and Schröter et al.^38, ^flows can also be distinguished by their spatial scale. Local flows occur when the supply and demand are located within the same spatial unit (e.g., a municipality), indicating that ecosystems directly support their immediate communities. Proximity flows arise when the supply is located near, but outside, the unit of demand, such as surrounding rural areas providing services to adjacent urban centers. Interregional flows, by contrast, refer to situations where flows span over long distances, often through infrastructure, markets, or information networks. While this study quantifies NCPs dynamics at the local (municipal) level, the resulting patterns of surpluses and deficits provide valuable insights into potential proximity and interregional dependencies. This enables discussion of broader spatial interconnections and governance implications, even from a local-scale lens. Nevertheless, we acknowledge that explicitly modeling these larger flows would offer deeper and more precise insights, particularly for analyses focusing on interregional or international scales. In such contexts, approaches like life-cycle assessment (LCA) are commonly used to trace material and environmental flows across regions and sectors. These frameworks offer a way to evaluate dependencies and externalized impacts associated with human-nature interactions^116,117^.

Expanding on the components listed in Table 1, three variables were derived for each municipality and each NCP (resulting in a total of 18 variables per municipality):

- The **current budget**, i.e., budget in the current timestep, offers an overview of the present conditions of the NCP in question. This corresponds to the most recent value for the component budget computed for each municipality and each NCP (Fig. 2 module A3.1).
- The **budget coefficient (B**_**coeff**_**)** represents the slope of a linear regression equation applied to budget values over four timesteps (Fig. 2 module B). For example, the budget coefficient of the NCP pollination is calculated by constructing a linear regression using the following values: 1980s POL_BUDGET_, 1990s POL_BUDGET_, 2000s POL_BUDGET_, current POL_BUDGET_. The slope of the resulting linear regression is referred to as the budget coefficient, which reflects the evolution of the budget for this NCP over time.
- The **ratio coefficient (R**_**coeff**_**)** similarly represents the slope of a linear regression equation applied to ratio values over four timesteps (Fig. 2 module B). This variable reflects how the percentage of demand met by supply changes over time.

The combination of the two coefficients (Fig. 2 module B) provides interesting observation points, namely, if a similar trend is observed for both the ratio and budget coefficients, it can be inferred that the development of the NCP in question is stable and that it will likely develop in a similar fashion in future years, if no policies or changes in societal actions influence this pattern. It is similarly possible for a municipality to exhibit a positive budget coefficient in combination with a neutral or negative ratio coefficient (or vice versa). This indicates a less stable and clear trend, which may develop into a positive situation or rather become increasingly problematic.

In a final step, these three variables (current budget, B_coeff_ and R_coeff_) were used in a clustering algorithm to identify patterns of change in spatiotemporal trends of NCPs demand and supply^29,118,119^ (Fig. 2 module C). Clustering approaches are particularly valuable for understanding how various factors vary across different regions, simplifying multidimensional data into meaningful groups that enhance interpretability and facilitate informed decision-making^118^. For this study, hierarchical clustering was chosen, due to its effective ability to successfully capture the underlying structure of the data^120^. The optimal number of clusters was determined by examining the hierarchical dendrogram (see Supplementary material D) and accounting for the specific characteristics of the case study, ultimately leading to the selection of four spatiotemporal clusters. This process was performed using the R programming language^121^ and the RStudio environment^122^and the clustering was specifically implemented with the factoextra package^123^.

### Case study: spatial and temporal reference

**Fig. 4:**
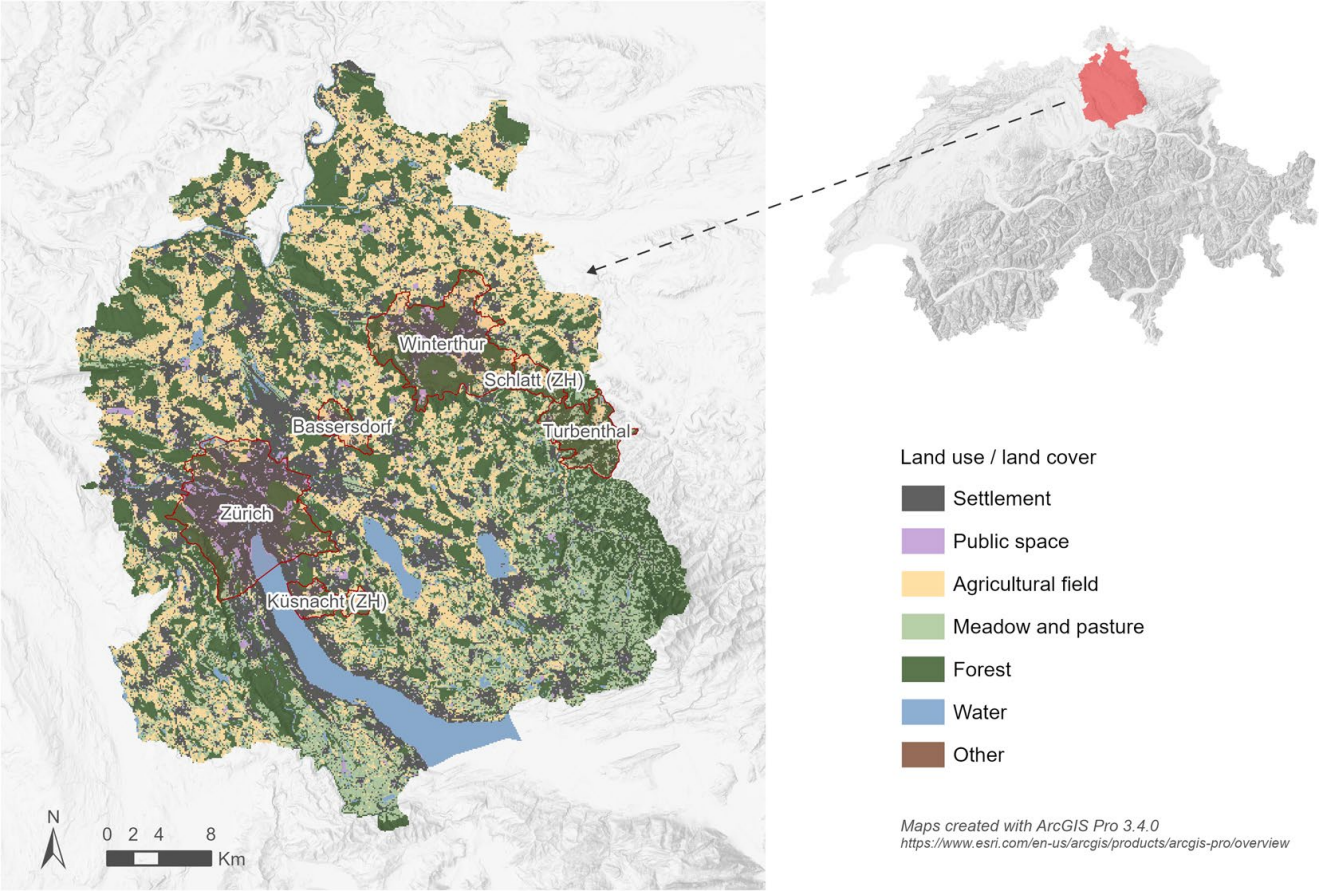
Overview map of the canton of Zurich, located in northeastern Switzerland. The land use land cover map shows seven main classes adapted from the Land use statistics standard nomenclature NOAS04 ^49^. Selected municipalities specifically discussed in this publication are highlighted and named. This and following maps were created with ArcGIS Pro 3.4.0 (https://www.esri.com/en-us/arcgis/products/arcgis-pro/overview).

This study focuses on the Canton of Zurich (Fig. 4), located in northeastern Switzerland and covering an area of approximately 1,730 km²^124^. The canton comprises a diverse mix of land uses, including forests, agricultural areas, settlements, and bodies of water. These varied landscapes contribute to the supply of multiple NCPS, such as climate regulation, food, and recreational spaces. At the same time, the growing urban and peri-urban population, which has increased around 60% over the past four decades^124 ^intensifies the demand for these NCPs. This evolving interaction between land use changes and population growth shows the relevance of choosing the canton of Zurich as a case study for this research.

## Results

### NCPs supply, demand, budget and ratio

**Fig. 5:**
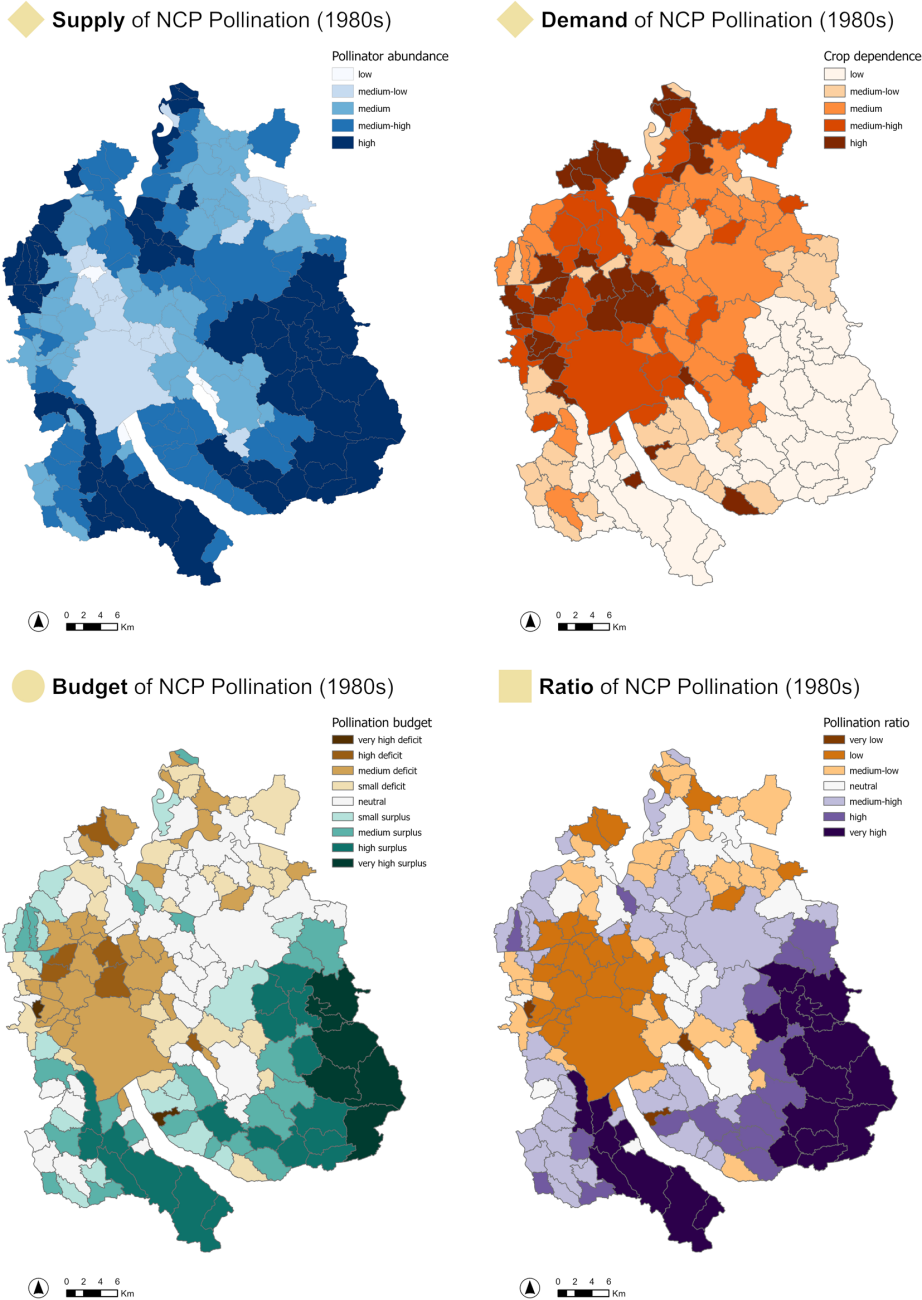
Spatial distribution of supply, demand, budget, and ratio for the NCP pollination in the canton of Zurich during the 1980s. The maps illustrate: (upper-left) pollination supply based on pollinator abundance; (upper-right) demand based on crop dependency; (lower-left) pollination budget (supply - demand); and (lower-right) ratio, i.e. the percentage of demand met by supply. In the methodological overview (Fig. 2) these outcomes correspond to one timestep in modules A2, A3.1 and A3.2. Similar maps for the other timesteps and NCPs are available in Supplementary material B).

For each NCP, 16 maps were generated, showing the components supply, demand, budget, and ratio across four timesteps (1980s, 1990s, 2000s, current). Figure 5 illustrates the results for the NCP pollination for the first timestep, 1980s. Similar maps were produced for all timesteps and all NCPs (Supplementary material B and Fig. 2 module A).

Overall, the spatial patterns of supply and demand vary considerably across NCPs and regions. While urban municipalities often display consistent mismatches, characterized by high demand and low supply, other areas exhibit more balanced or fluctuating patterns, depending on the specific NCP and land use. Less densely populated areas or those with a greater natural character in the south-east tend to have higher supply levels, whereas demand often concentrates in municipalities with intensive land use or larger populations in the center-west of the canton. These patterns, where urban municipalities exhibit high demand and low supply, and more rural areas present a surplus, is consistent with prior findings that highlight urban reliance on external ecosystem benefits^18,19, and ^emphasize the need for coordinated place-specific policy responses by revealing clear spatial NCPs mismatches.

### NCPs trends and perspectives

**Fig. 6:**
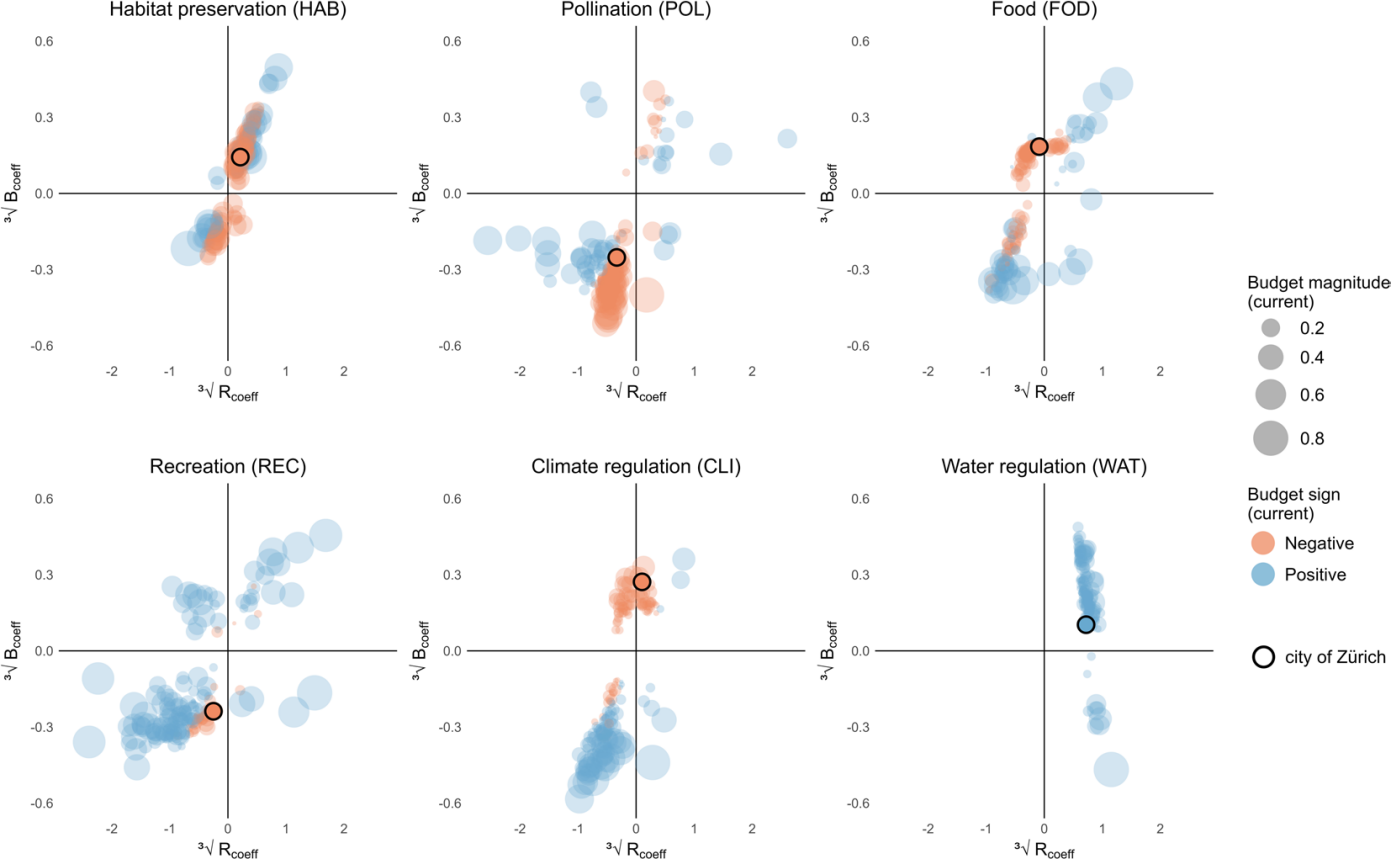
Temporal trends in NCPs budget and ratio for municipalities in the canton of Zurich. Each dot represents a municipality, showing the relationship between the budget coefficient (B_coeff_) and the ratio coefficient (R_coeff_) for selected NCPs (habitat preservation, pollination, food, recreation, carbon regulation, and water regulation). The size of the dots corresponds to the budget magnitude for the current timestep, while the color indicates the sign (blue = positive, red = negative). The city of Zurich is marked with a black border. To improve interpretability, the coefficient values and their corresponding axes have been cube-root-transformed. In the methodological overview (Fig. 2) these temporal trends correspond to module B (and partly A3.1).

When temporal dynamics are added, these spatial NCPs patterns become more complex due to changes in supply and demand over time. This complexity further emphasizes the need for our proposed integrative approach, which combines both spatial and temporal perspectives to better understand human-nature interactions and inform sustainable resource management. Figure 6 illustrates the long-term trends of the budget and ratio coefficients (B_coeff_ and R_coeff_), as well as the current budget for each municipality in the canton of Zurich (Fig. 2 module B). Each dot represents a municipality in the canton of Zurich and shows its status and trends for a specific plot, i.e. NCP. The color of the dots indicates the current NCP budget: blue for a positive current budget and red for a negative current budget. The size of each dot reflects the magnitude of this most recent budget. The x-axis (R_coeff_) shows the direction and strength of the change in the NCP ratio over time: positive values indicate an increase in the NCP ratio, while negative values a decrease. The y-axis (B_coeff_) shows the trend in the NCP budget over time: values above zero reflect increasing NCP budgets, while values below zero indicate a decline. A detailed guide on interpreting these plots is provided in Supplementary material C.

The city of Zurich, highlighted in each plot by a black-bordered dot, illustrates a predominantly negative NCPs situation. Most NCPs show moderate negative current budgets, with pollination and recreation displaying consistently declining trends over time. For habitat preservation, food provision, and climate regulation, this municipality exhibits mixed and unstable trends over time, with only water regulation showing a positive trend (and positive current budget). Overall, the city’s NCPs situation points to persistent deficits in the delivery of several key NCPs, with only minor positive trends, underscoring the need for targeted interventions to address these spatial mismatches. However, while individual patterns such as those described for the municipality of Zurich can be clearly identified, our assessment offers also the possibility of focusing on broader dynamics and overarching developments.

Across all NCPs, the proportion of municipalities showing positive trends in both budget and ratio coefficients varied considerably. Water regulation has the highest proportion, with 97% of municipalities showing positive past trends. Similarly, 72% of municipalities showed past improvements in the NCP habitat quality. In contrast, pollination and recreation present the lowest shares of municipalities, with only 17% and 19%, respectively, displaying positive trends. Finally, a moderate number of municipalities exhibit negative past trends in food provision (39%) and carbon sequestration (32%). Another key observation from Fig. 6 is that municipalities with positive current budgets exhibit greater variability in NCPs supply and demand (as indicated by larger values of B_coeff_ and R_coeff_). These regions currently enjoy a surplus of these specific NCPs, but they were also subject to more dynamic shifts in the past. In contrast, municipalities with negative budgets (red dots) across several NCPs, including habitat preservation, recreation, food, and to a certain extent climate regulation, tend to display more stability and slower changes over time (smaller B_coeff_ and R_coeff_ values). Figure 6 also indicates a substantial degree of variability across municipalities within the canton, with no general discernible pattern emerging either for all municipalities or across different NCPs. Instead, a complex configuration emerges, with municipalities exhibiting a wide range of responses to changing environmental and societal conditions. An exception to this is the NCP water regulation, where all municipalities consistently exhibit positive current budgets, though with differences in the budget coefficients. In general, these variations highlight the uneven conditions across NCPs and underscore the need for targeted interventions where negative trends are prevailing.

#### Spatiotemporal NCPs clusters

**Fig. 7:**
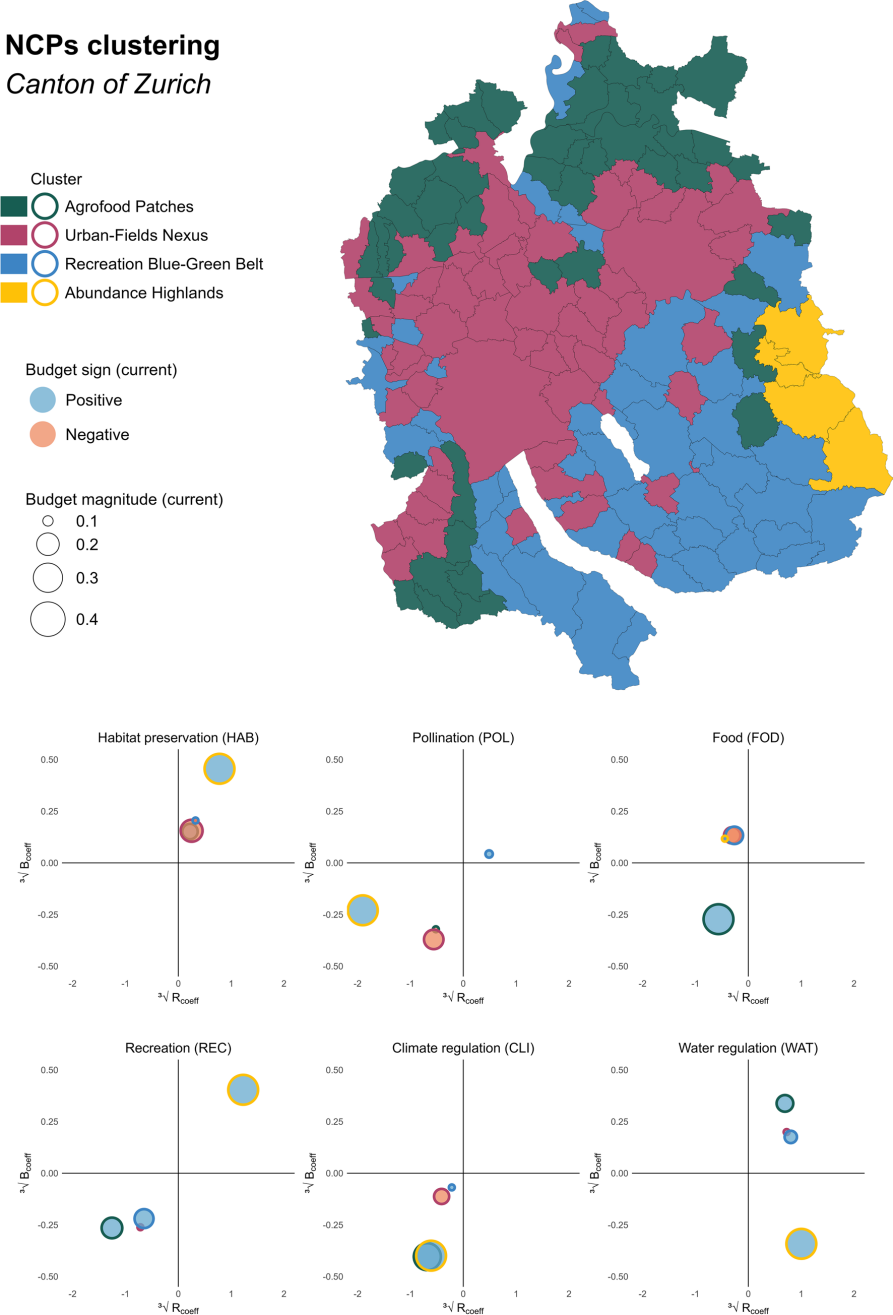
NCPs clustering in the canton of Zurich. The map in the upper part of the figure shows the clustering of municipalities based on NCPs information. Each color represents a different cluster, indicating municipalities with similar NCPs characteristics. The colors of the clusters on the map match the colors of the dots. The six bubble-plots in the lower part of the figure illustrate NCPs budget and ratio coefficients for the four clusters (average values of all municipalities in the respective cluster). For a detailed description see Fig. 6. In the methodological overview (Fig. 2) this clustering approach corresponds to module C.

In terms of policy and management, identifying common patterns among municipalities is particularly valuable, as it can guide coordinated responses to shared challenges. To this end, we used a clustering algorithm to group municipalities with similar spatiotemporal NCPs trends. Figure 7 illustrates the outcomes of the clustering analysis, highlighting the characteristic NCPs configurations of each of the four identified groups. Despite geographic location not being used as an input variable, the clusters display clear spatial patterns and distinct differences in land cover composition (see Supplementary Material E). Comparable grouping approaches, such as studies focusing on bundles^22,25,32^ or archetypes^30 ^have been used to characterize patterns in other regions and contexts^25,30,125^. Our approach builds on this established research and expands it by incorporating an in-depth understanding of the spatiotemporal trends of NCPs supply and demand.

The **Urban-Fields Nexus** cluster (dark red), which includes cities like Zurich, Winterthur, and their surrounding municipalities, is characterized by a high density of settlements and infrastructure, along with significant – though less extensive compared to other clusters – forest and meadow areas. This cluster also has the largest share of parks and public spaces, emphasizing its urban character, and a considerable portion of land is allocated to agriculture and horticulture. In terms of NCPs, the Urban-Fields Nexus cluster shows a negative budget for habitat preservation (HAB_BUDGET_), but positive past trends for both coefficients (B_coeff_ and R_coeff_). The NCP pollination reveals a more concerning picture, with negative current budget (POL_BUDGET_), and trends indicating a growing deficit. Food provision and water regulation currently display positive budgets (FOD_BUDGET_ and WAT_BUDGET_) with positive historical development, while the NCPs recreation and climate regulation also present modest surpluses (REC_BUDGET_ and CLI_BUDGET_), but their past evolution shows declining trends. These findings are relevant also to other urbanizing contexts aiming to reconcile dense settlement development with ecosystem functionality.

A more comprehensive understanding of the spatial and temporal trends of municipalities can be achieved by identifying which cluster they belong to. For instance, despite not being in close geographical proximity, the municipalities of Zurich, Küsnacht, and Bassersdorf all belong to the Urban-Fields Nexus cluster and share similar current situations and historical trends in the supply of and demand for NCPs (Supplementary material F). In the current timestep, all three exhibit positive current budget for water regulation (WAT_BUDGET_) and negative budgets for habitat preservation (HAB_BUDGET_), pollination (POL_BUDGET_), food (FOD_BUDGET_), and climate regulation (CLI_BUDGET_), indicating that demand exceeds supply. The historical trajectories of these NCPs vary, some indicating improvement and others deterioration, suggesting that future outcomes may either reinforce current imbalances or offer opportunities for recovery, depending on the specific NCPs and the persistence of past trends. An interesting case emerges with the NCP recreation. Here, the three municipalities do not share similar current budgets – Küsnacht has a budget surplus – though they follow a common historical trend. Considering the characteristics of Küsnacht and Bassersdorf, both situated in the Zurich agglomeration area, it is possible that these municipalities are experiencing similar influences and pressures^85,126,127^. However, Küsnacht landscapes provides a more robust supply of recreational spaces, compared to Bassersdorf. Despite this advantage, the shared historical trends suggest that, without intervention to either increase the supply or manage the demand for recreational spaces, Küsnacht may gradually shift toward the same NCPs constellation as Bassersdorf, and later Zurich, and the recreational budget could decline from surplus to neutral or even negative^126^. This example illustrates the value of cluster-based analysis in revealing common patterns of development and pressure among municipalities that may not be immediately apparent from spatial proximity or from their sociodemographic characteristics alone.

The municipalities within the **Recreation Blue-Green Belt** cluster (blue) are mainly situated in the southern and central-eastern regions, particularly around and between the lakes of Zurich, Greifensee, and Pfäffikon. This cluster is characterized by a balance between infrastructure, forest and agricultural areas, and an important presence of meadows and pastures, underlining the importance of natural and extensive agricultural landscapes in these areas. Regarding NCPs, the Recreation Blue-Green Belt cluster shows a positive current budget with upward historical trends (B_coeff_ and R_coeff_) for water regulation (WAT_BUDGET_). Recreation also displays a positive current budget (REC_BUDGET_), but declining trends signal a progressive deterioration. A similar scenario applies to pollination, where a positive current budget (POL_BUDGET_) is linked to contrasting past developments. Climate regulation shows a small negative current budget (CLI_BUDGET_) with a negative past trend that progressively worsened the balance between supply and demand. Habitat preservation has a similar small negative current budget (HAB_BUDGET_), but positive past development suggests that this situation has been gradually improving. Finally, the NCP food exhibits a considerable negative current budget (FOD_BUDGET_), yet the contrasting historical development (B_coeff_ and R_coeff_) introduce uncertainty on whether the balance for this NCP is strengthening or rather weakening.

The **Abundance Highlands** cluster (yellow) is situated in the hilly regions in the south-eastern part of the canton. The municipalities in this area are distinguished by extensive forest cover as well as meadows and pastures, but sparse infrastructure, indicative of the cluster’s natural and rural character. The Abundance Highlands cluster exhibits considerable positive budgets for all NCPs, but the NCP food essentially shows a neutral balance between supply and demand (FOD_BUDGET_). This general positive situation reflects the natural and rural character highlighted above. The favorable trends (B_coeff_ and R_coeff_) in habitat preservation and recreation indicate past improvements for these NCPs, while the negative trends for pollination and climate regulation hint at historic progressive deterioration. Regarding the NCPs food and climate regulation, the opposing past trends (B_coeff_ and R_coeff_) do not indicate a clear development.

The **Agrofood Patches** cluster (dark green) is distributed across large patches of municipalities in the southwestern and northern regions of the canton. These municipalities are characterized by large areas of intensive agriculture and horticulture, as well as a balanced mix of forests, meadows, and pastures. This diverse landscape structure is reflective of the varied land uses observed across the municipalities in question. Most NCPs in the Agrofood Patches cluster exhibit favorable current budgets. However, only habitat preservation (HAB_BUDGET_) and water regulation (WAT_BUDGET_) display positive past trends (B_coeff_ and R_coeff_), indicating a persistent improvement of the situation in the past. Positive budgets are evident for food provision (FOD_BUDGET_), recreation (REC_BUDGET_), and climate regulation (CAR_BUDGET_), although these are accompanied by negative past trends (B_coeff_ and R_coeff_). Finally, pollination (POL_BUDGET_) is the only NCP with a small negative current budget, which is further associated with a negative historical development.

Besides describing municipalities within the same cluster, these outcomes can be also used to compare municipalities that are geographically close but belong to different clusters. For example, Winterthur, which is part of the Urban-Fields Nexus cluster, has negative NCP food values, which have remained stable over time. In contrast, the neighboring municipality of Schlatt (Agrofood Patches) has a positive current budget but a negative historical trend. Considering *proximity* can be beneficial when evaluating the present situation and potential future implications. Winterthur has consistently had a negative food budget, indicating its reliance on surplus from other municipalities and regions to meet demand. In contrast, Schlatt’s positive current food budget has diminished over time. This decline can be attributed to changes within the municipality as well as pressure from neighboring urban areas and resulting NCPs proximity flows. A different dynamic emerges for NCP recreation. Winterthur has a negative budget, while neighboring municipalities, such as Turbenthal (Abundance Highlands), have positive budgets. This suggests a NCP proximity flow, whereby residents from urban areas (e.g., Winterthur) seek recreational opportunities in neighboring municipalities with a supply surplus. This example as well as the one mentioned above focusing on the Urban-Nexus cluster demonstrate how clusters can characterize municipalities and their NCPs trends and show how local-level data can be used to discuss *proximity* or *interregional* NCPs flows at larger scales (Supplementary material F).

## Discussion

This study provides a thorough integration of NCPs supply and demand assessments over space and time. Building on previous attempts to combine and synthesize some of the diverse facets of NCPs assessments^19,35, ^our methodology integrates temporal trends (B_coeff_ and R_coeff_) with present conditions (current budgets), enabling the recognition of current trade-offs and potential developments that can inform management strategies. Moreover, since municipalities frequently exhibit analogous development over time, identifying and capturing these can help prioritize effective strategies to secure the long-term supplied and demanded NCPs. To achieve this, the present study expands on existing research on clustering approaches^31,125^ by incorporating both present conditions as well as historical trends. The clustering procedure provided a powerful tool for understanding the multifaceted roles that landscapes play over time and highlighting areas where certain services may be at risk and where synergies or trade-offs between different NCPs might occur^30,33,125,128^. The insights gained from this approach emphasize the necessity for management strategies that are sensitive to patterns of stability and fluctuation, to facilitate the identification of common developments and the implementation of tailored strategies.

The conceptual approach of this study (see also Fig. 1) focuses on spatiotemporal trend of NCPs supply and demand at the *local* level – with NCPs flows occurring within the boundaries of a single municipality – providing a strategic and practical lens for assessing how municipalities meet their own NCPs demands. This choice is particularly relevant since municipalities serve as key entities for management and policy decisions, making them suitable units for the analysis of supply-demand dynamics and the elaboration of management strategies^126,129^. It is, however, important to recognize that certain NCPs, such as pollination, climate regulation or habitat preservation, exhibit spatial heterogeneity even within a single municipality^52, ^yet these sub-local flows and variations were not incorporated into the analytical framework of this study. For instance, using Net Primary Productivity (NPP) as a proxy for climate regulation services does not fully capture complex carbon dynamics. Similarly, widely implemented models like InVEST depend on assumptions that may not perfectly reflect local ecological conditions. Nevertheless, while our approach inevitably overlooks fine-scale ecological variations, it was deemed appropriate and necessary to assess the long-term evolution of NCPs at the municipal scale in a consistent and coherent way. Thus, despite this limitation, our findings offer valuable insights into *local* flows and discrepancies between supply and demand for individual municipalities. However, even if this study does not directly model *proximity* and *interregional* flows, nor does it rely on origin-destination data, the spatial patterns of *local* supply and demand still allow for indirect interpretations about broader patterns of dependence. In fact, municipalities with budget deficits, and particularly those with negative past trends, can only meet their demands through the supply of municipalities with NCPs surpluses, demonstrating a necessity for compensation through *proximity* or *interregional* flows. Yet, this dynamic does not apply equally to all NCPs. The flows of NCPs can vary significantly depending on the nature of the resource in question. For example, the NCPs water regulation and food are strongly influenced by external environmental and even economic factors, revealing the potential for regional measures (i.e. between groups of municipalities) to achieve neutral or even positive NCPs budgets^130^. Conversely, some NCPs, such as pollination, are more dependent on local biophysical conditions and may not be as easily compensated for by neighboring or more distant municipalities^52^. These insights into NCPs flow dynamics can guide policies that aim to balance local self-sufficiency with regional interdependence. As illustrated by other studies^19,38, ^by establishing frameworks that support *local*, *proximity*, and *interregional* flow adaptations, municipalities can address localized NCPs shortfalls while also leveraging the strengths of neighboring areas to support their *local* needs. The scope can be further extended by considering broader, international NCPs flows, each with unique scale and implications^12^. An urban municipality such as Winterthur only partly satisfies its NCPs budgets with flows from neighboring municipalities. For instance, the NCPs climate and water regulation rely on biophysical flows that inherently transcend national borders, impacting landscapes and populations on a broader scale. Other NCPs, such as food, are influenced by the movement of commodities, whereby trade connects diverse landscapes through local, regional, but also global supply chains. The recognition of these international flows, whether driven by biophysical processes or economic demands, emphasizes the interconnectedness of NCPs^6^. Future research could expand our methodology by delving into these international flows, exploring how various NCPs interact across time and national borders and examining the implications of these flows for global sustainability and ecosystem resilience, to understand how to best manage NCPs in a deeply interconnected world.

The outcomes of our spatiotemporal NCPs analysis underscore the necessity of governance approaches that are tailored to multi-level, polycentric challenges rather than relying on uniform policy solutions^42^. Each municipality exhibits distinct socio-environmental conditions, reinforcing the need for governance structures that are adaptive to spatial heterogeneity and capable of integrating diverse stakeholders across different levels of decision-making. At the same time, the identification of clusters highlights the potential for collaborative governance mechanisms, allowing municipalities facing similar pressures – such as those within the Recreation Blue-Green Belt – to align their management strategies and co-manage NCPs budgets efficiently^6,19^. Recognizing the interplay between localized specificities and regional interdependencies is critical for designing governance frameworks that balance autonomy with cooperative action, ensuring that policies remain flexible and responsive to evolving socio-environmental conditions. Our findings align with polycentric governance theory^42,43, ^which emphasizes the need for nested, adaptive decision-making across scales rather than imposing uniform governance models. Our methodology is particularly well-suited to diagnose multi-level governance challenges by identifying governance gaps and opportunities at local, regional, and national levels, moving beyond static governance prescriptions. Instead of a panacea approach, which assumes a single governance system (e.g., centralized planning, privatization, or community-based management) can resolve all environmental challenges, our methodology supports a context-sensitive, adaptive management approach that tailors governance solutions to specific social-ecological conditions. In this context, the Swiss federalist system serves as an illustrative example of how multi-tiered governance structures can balance decentralization with strategic coordination, ensuring that environmental policies remain both empirically grounded and adaptable to local realities^131–133^. Planning instruments such as Communal, Regional, and Cantonal Structure Plans demonstrate how governance mechanisms can be differentiated across levels while maintaining coherence through institutional linkages^47,134,136^. This nested governance structure allows for self-organization, local experimentation, and institutional learning – key elements identified as essential for resilient environmental governance^42,43^. Beyond its immediate implications for the Swiss context, our findings also contribute to broader discussions on sustainability governance. Our attempt at aligning spatiotemporal supply and demand of NCPs offers a replicable methodology for managing these services in diverse settings, particularly those exhibiting similar urban-rural dynamics and decentralized planning systems. Future research should continue refining approaches to enhance institutional adaptability and ensure that governance models effectively integrate both local autonomy and coordinated action across scales^42,137^.

## Conclusion

This study incorporates complementary analyses to evaluate the spatial and temporal dynamics of NCPs at the local level, thereby promoting sustainable landscape development. Our approach combines historical trends with current conditions to detect emerging alignments and mismatches and recognize related consequences. This dual perspective on past and present conditions is crucial to identify current and predict future resource conflicts and to promote proactive management.

Applied to municipalities in the canton of Zurich, our approach serves to identify areas with persistent deficits in specific NCPs, as well as municipalities that currently function as net providers. In all municipalities, fluctuations in supply and demand over time highlight the dynamic nature of these interactions. The clustering analysis shows how municipal trajectories can align or diverge in response to land-use change, urban growth, and demographic pressures. These findings highlight the complexity of these interactions, but also demonstrate that the approach proposed by this study is relevant and adaptable to other contexts where dynamic changes necessitate adaptive management. While our assessment focuses on local scale and flows, the outcomes also point to spatial dependencies: municipalities with consistent deficits depend on others with surpluses, while provider municipalities may face increasing pressure if current trends continue, indicating that historically stable landscapes may be susceptible to overexploitation or competing demands if these trends persist. Expanding this approach to include cross-regional flows and more detailed ecological modeling would offer further insights into how supply and demand interact at broader scales.

Ultimately, this spatiotemporal assessment offers a valuable opportunity for identifying supply-demand mismatches and supporting adaptive governance. Moving beyond static one-size-fits-all approaches, the findings reinforce the need for governance strategies that account for spatial interdependence and dynamic developments. By integrating these principles, planning processes can become more responsive, coordinated and support sustainable human-nature interactions.

## Supplementary Information

Below is the link to the electronic supplementary material.

Supplementary Material 1

## Data Availability

The datasets used and analyzed during the study are available from the corresponding author on reasonable request.
