# Supplementary material for: Aligning spatiotemporal supply and demand of nature’s contribution to people (NCPs) for sustainable resource management
Source: Sci Rep. 2025 Sep 11;15:32412. doi: 10.1038/s41598-025-17652-4 (PMC12426217; doi:10.1038/s41598-025-17652-4)
Supplement: Supplementary file 1 — Supplementary Material 1 [file 41598_2025_17652_MOESM1_ESM.pdf]

Aligning spatiotemporal supply and demand of Nature's Contribution to People (NCPs) for sustainable resource management

Supplementary material

A. Qualitative contents analysis: Relevant planning documents

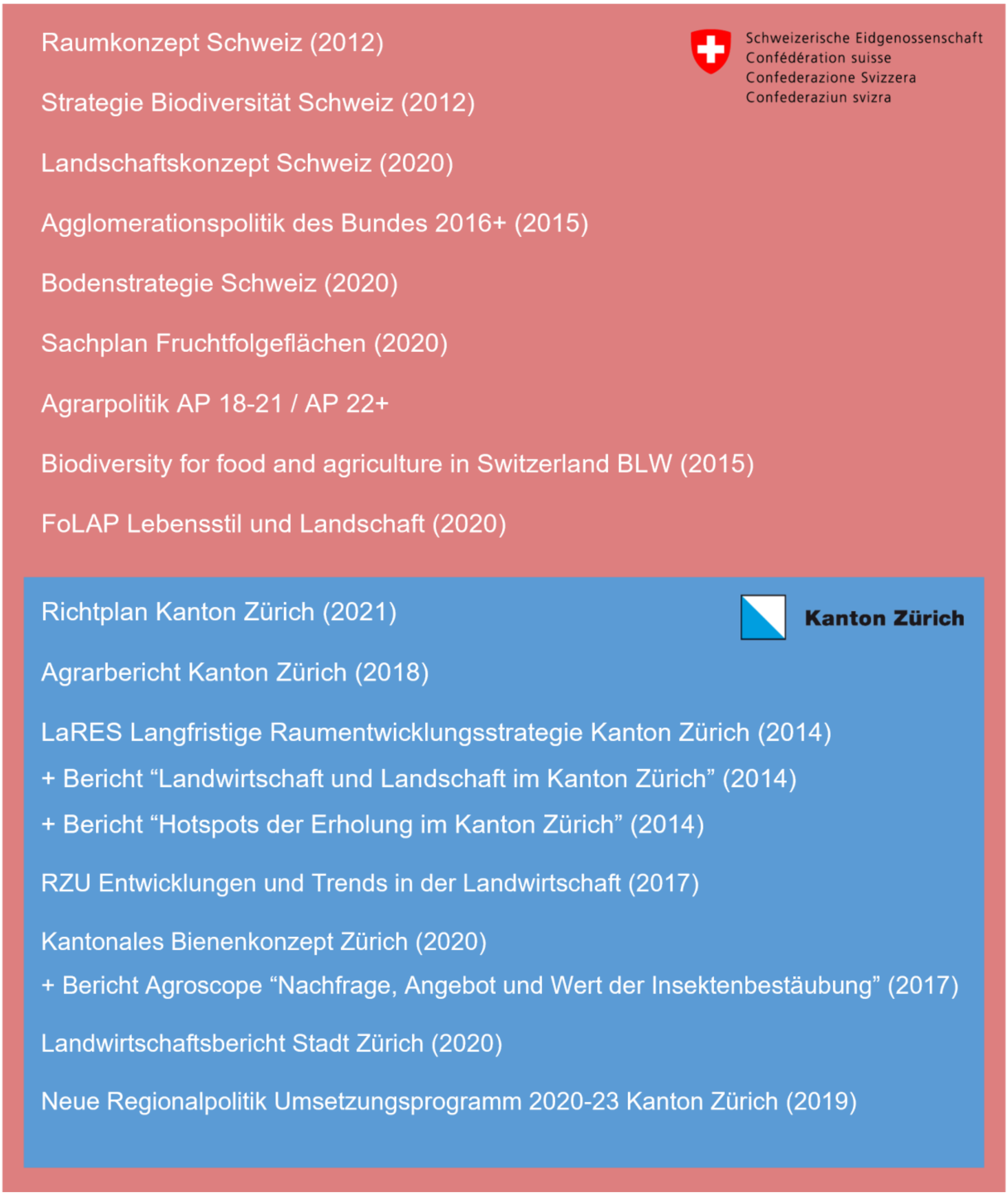

B. Supply, demand budget and ratio maps of each NCP

In the methodological overview (Figure 2 in the manuscript) these outcomes correspond to module A2, A3.1 and A3.2. For the legend of all the following maps please refer to the maps included in the main manuscript.

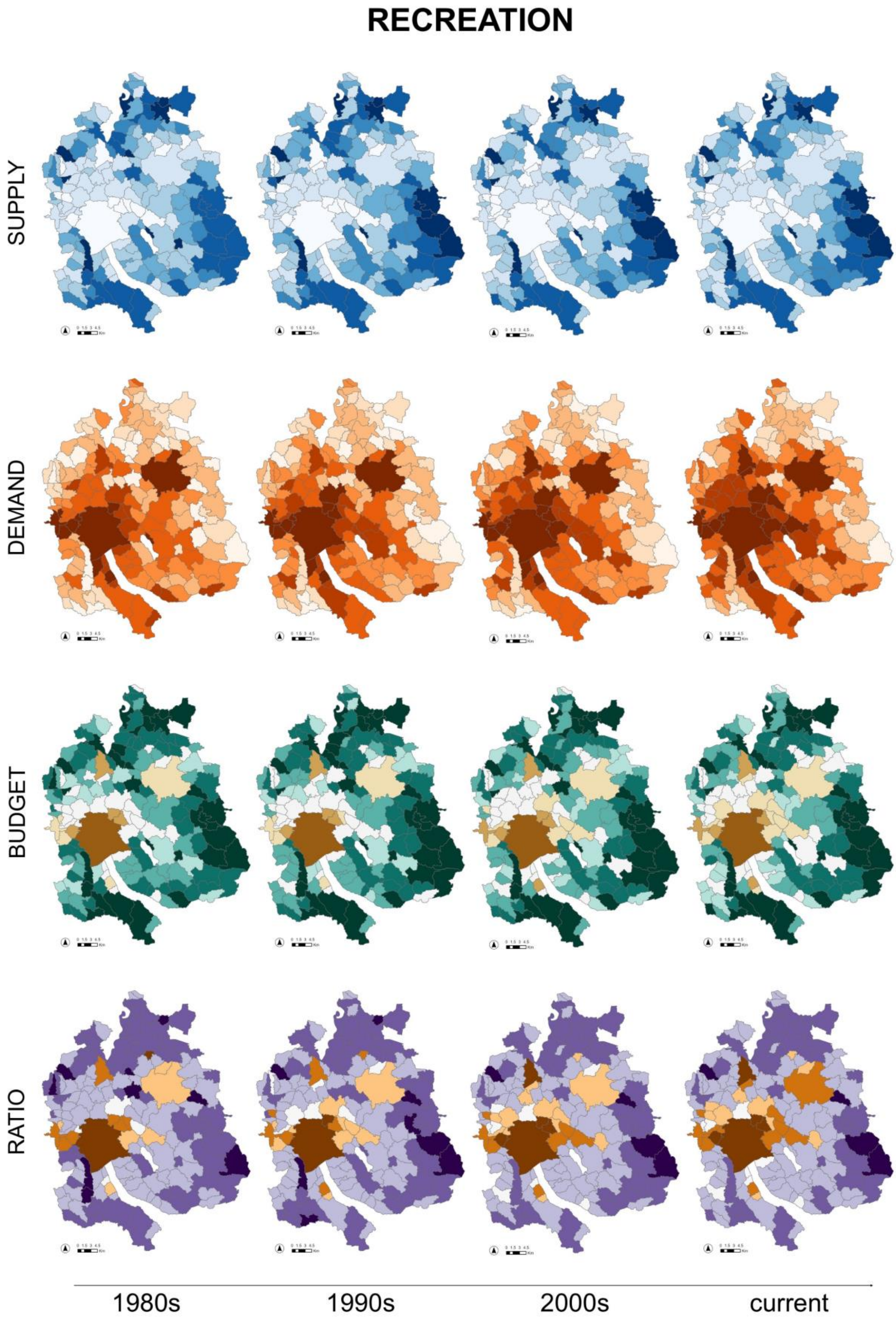

WATER REGULATION

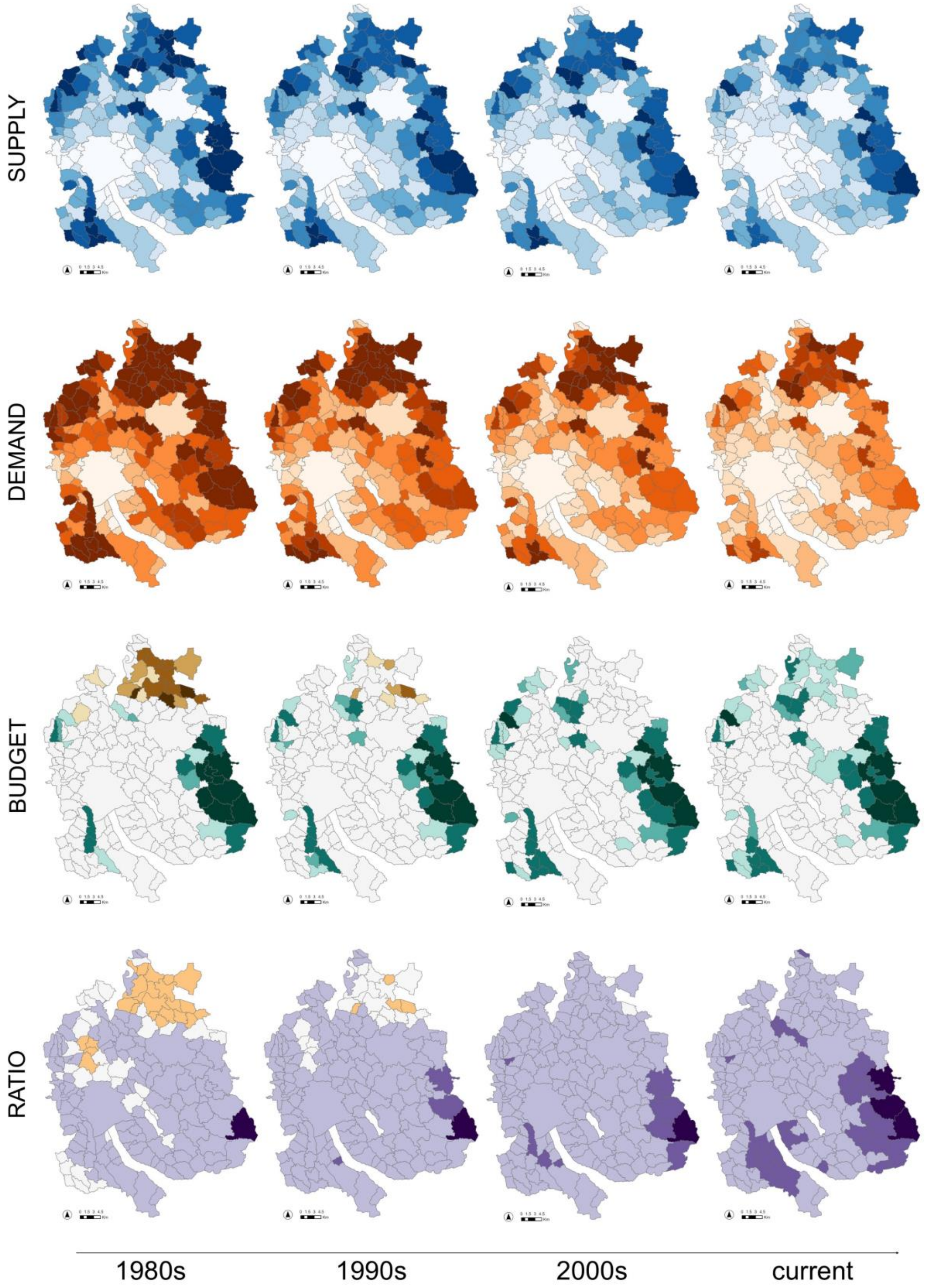

CLIMATE REGULATION

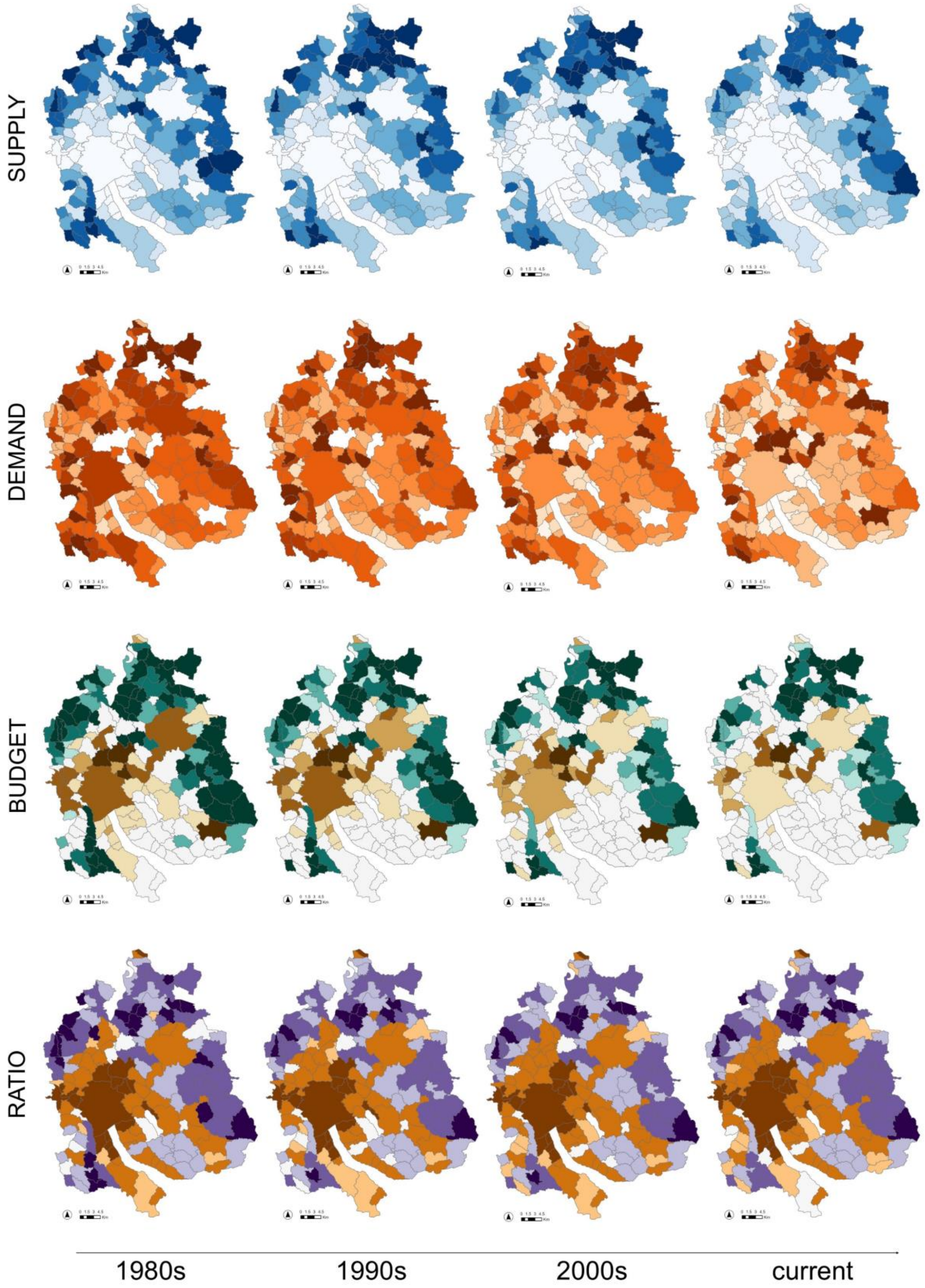

FOOD

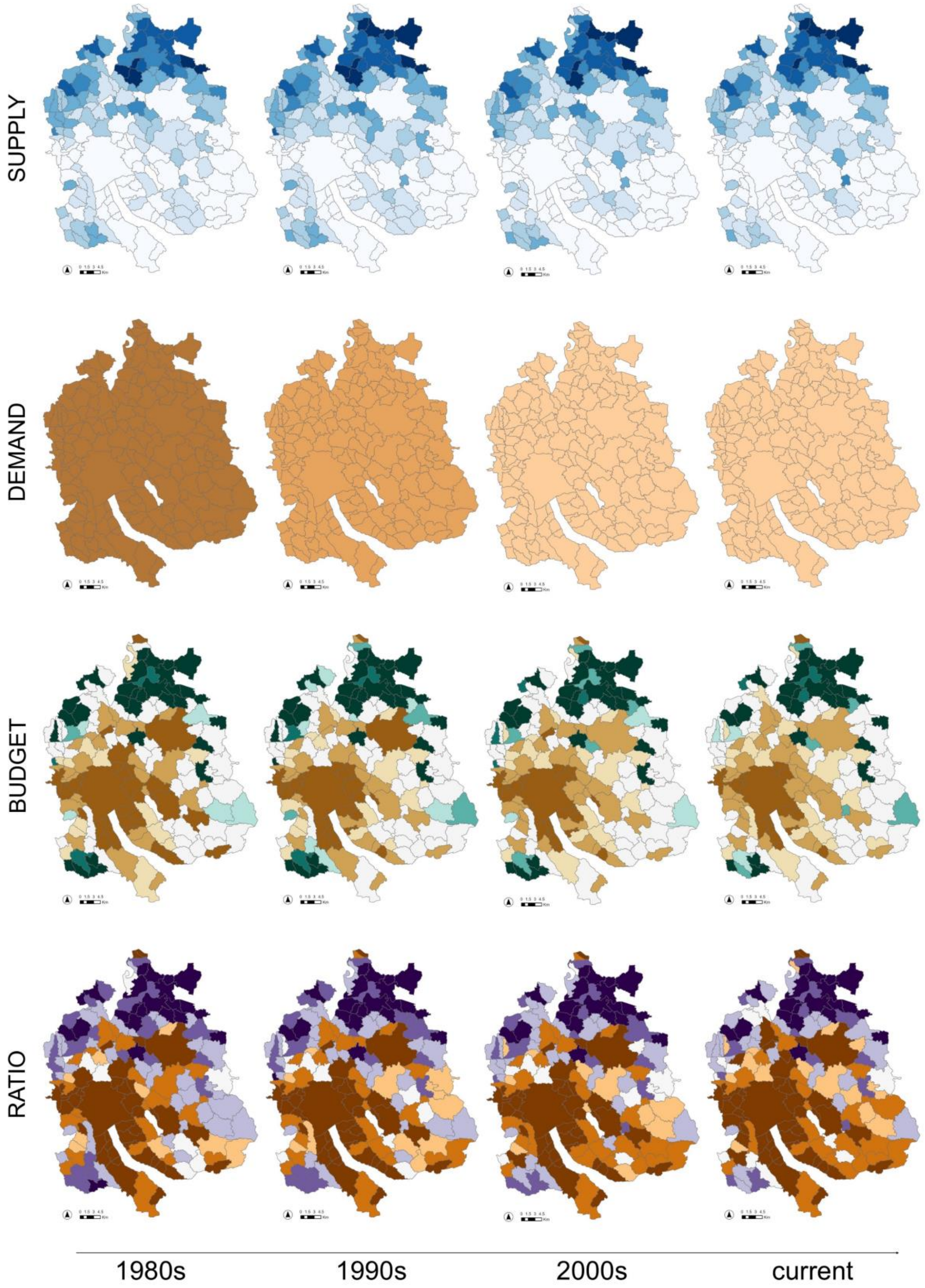

HABITAT PRESERVATION

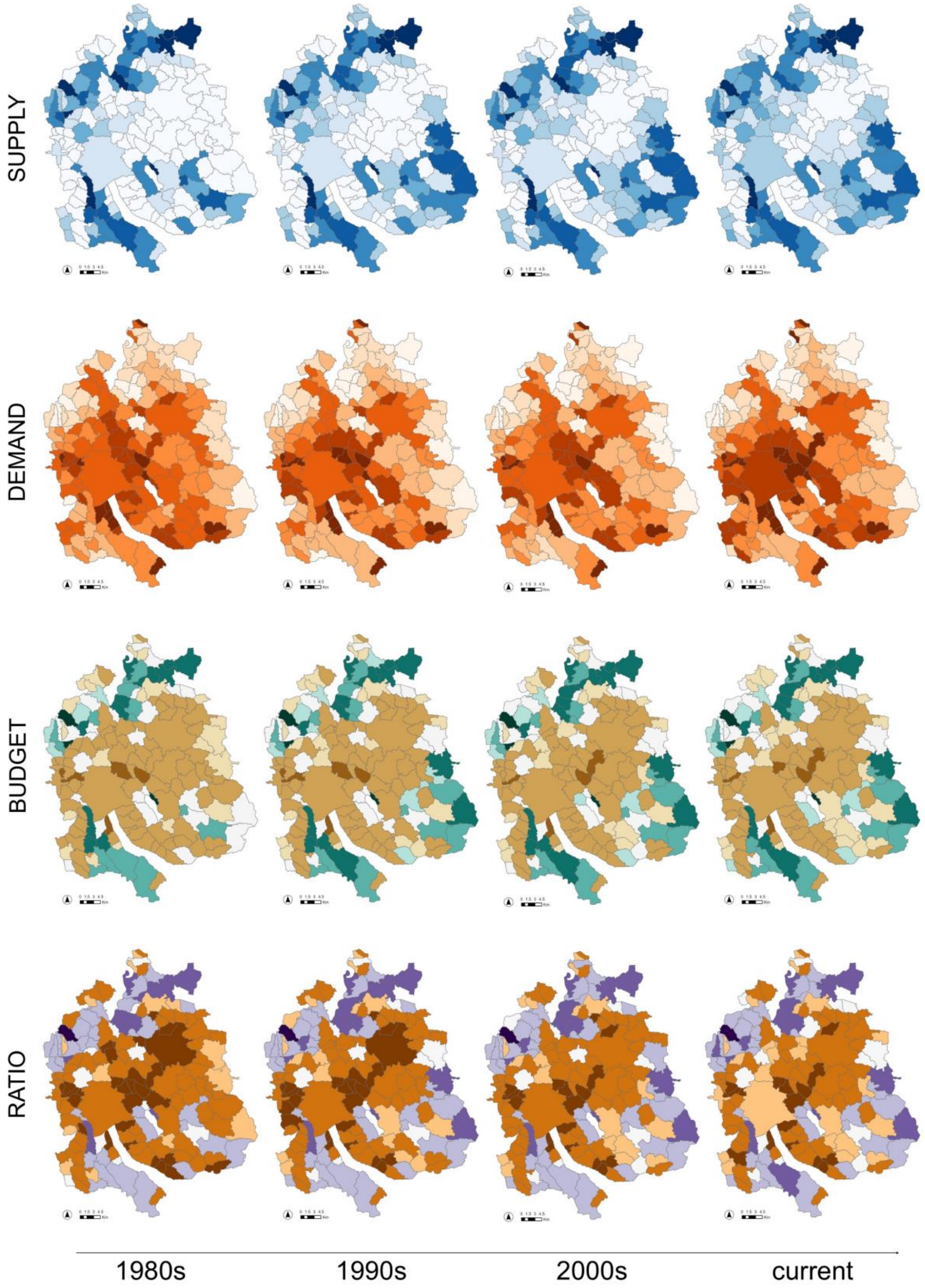

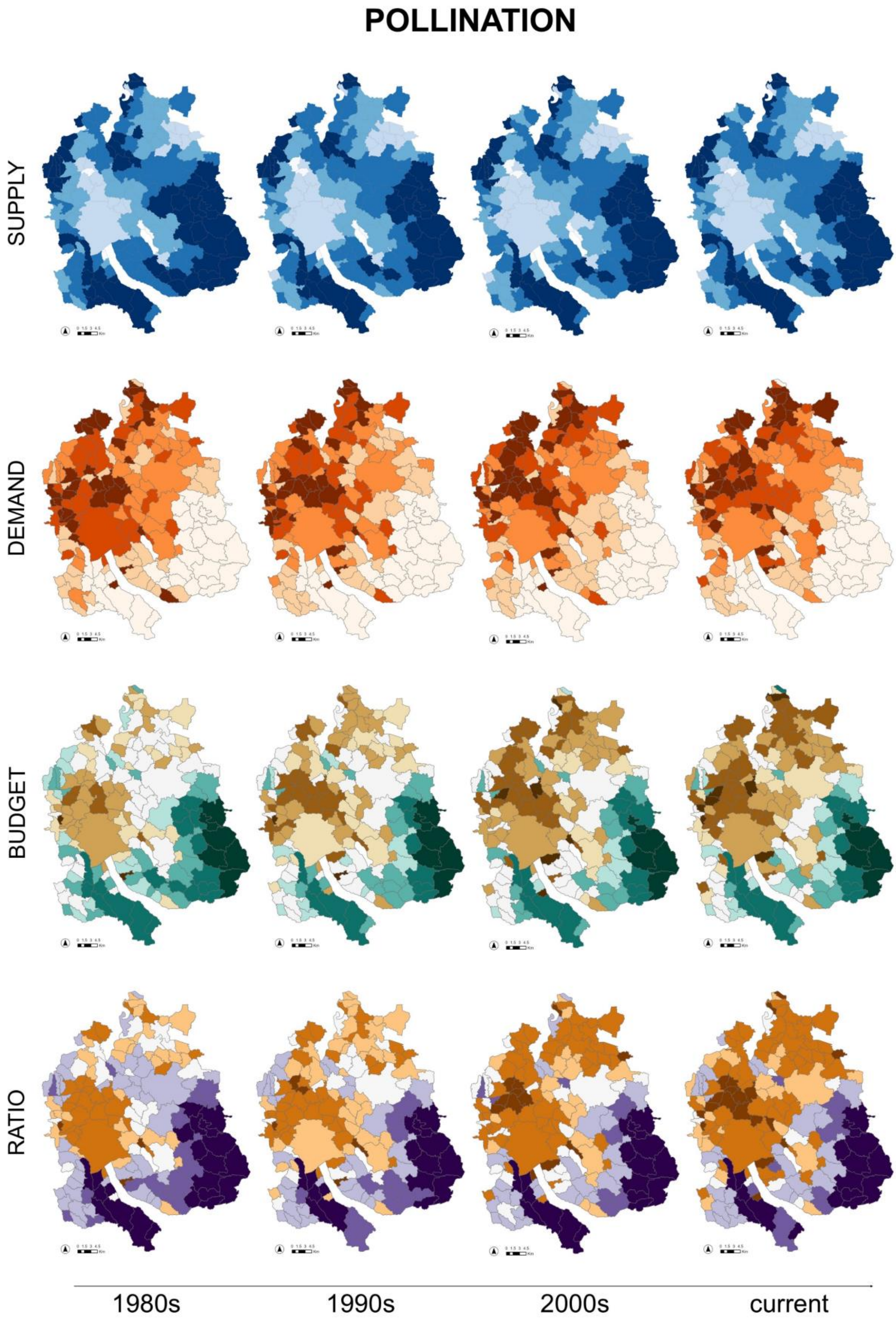
POLLINATION
SUPPLY
DEMAND
BUDGET
RATIO
1980s
1990s
2000s
current

15

C. Walk-through – Temporal trends in NCP budget and ratio

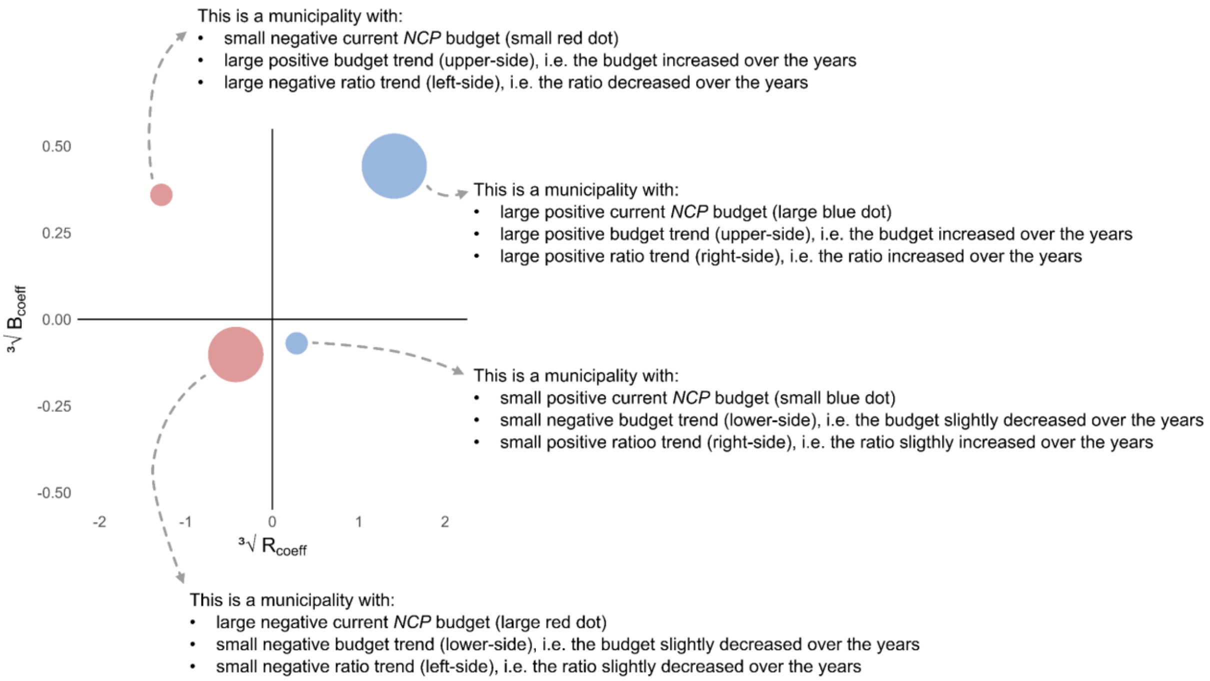

Figure C1: Detailed, walk-through of the temporal trends in NCPs budget and ratio for municipalities in the canton of Zurich. Each dot represents a municipality, showing the relationship between the budget coefficient (B_{coeff}) and the ratio coefficient (R_{coeff}) for selected NCPs (habitat preservation, pollination, food, recreation, carbon regulation, and water regulation). The size of the dots corresponds to the budget magnitude for the current timestep, while the color indicates the sign (blue=positive, red=negative). All the coefficients are cube-root transformed.

D. Dendrogram of hierarchical clustering

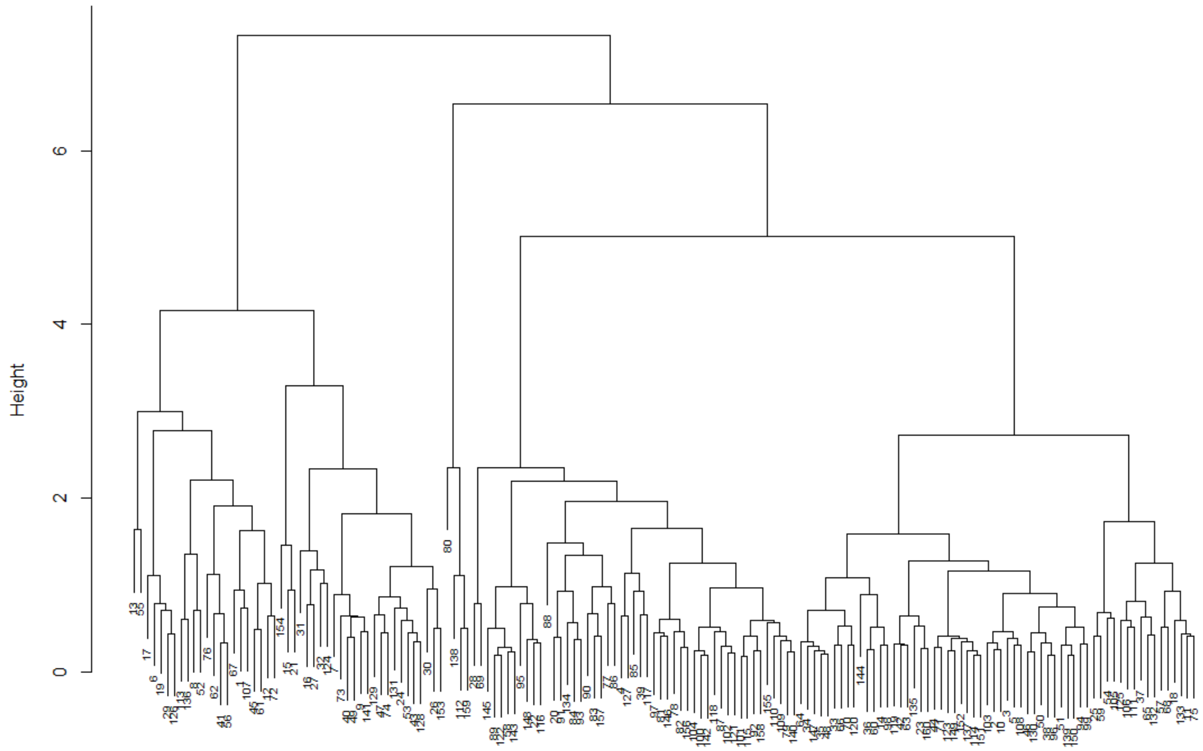

Figure D1: 3D scatter plot showing the distribution of data points across three principal components, with points color-coded to represent the four resulting clusters from the hierarchical clustering analysis.

E. Land use and land cover analysis of the NCPs clusters

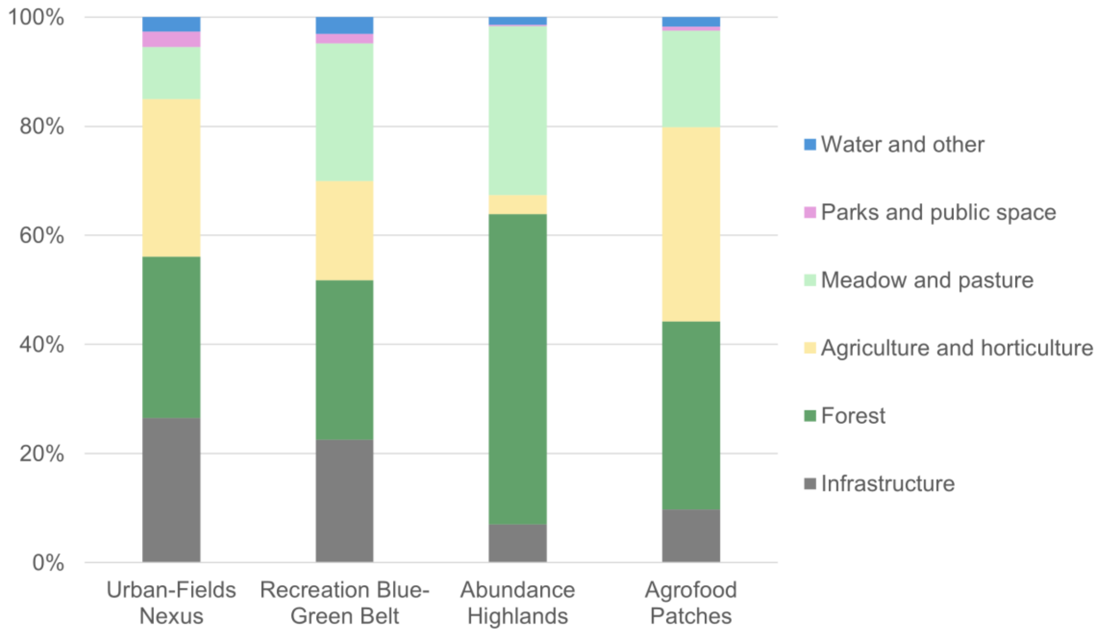

Figure E1: Land use and land cover distribution across NCPs clusters.

F. Temporal trends in NCPs for selected municipality

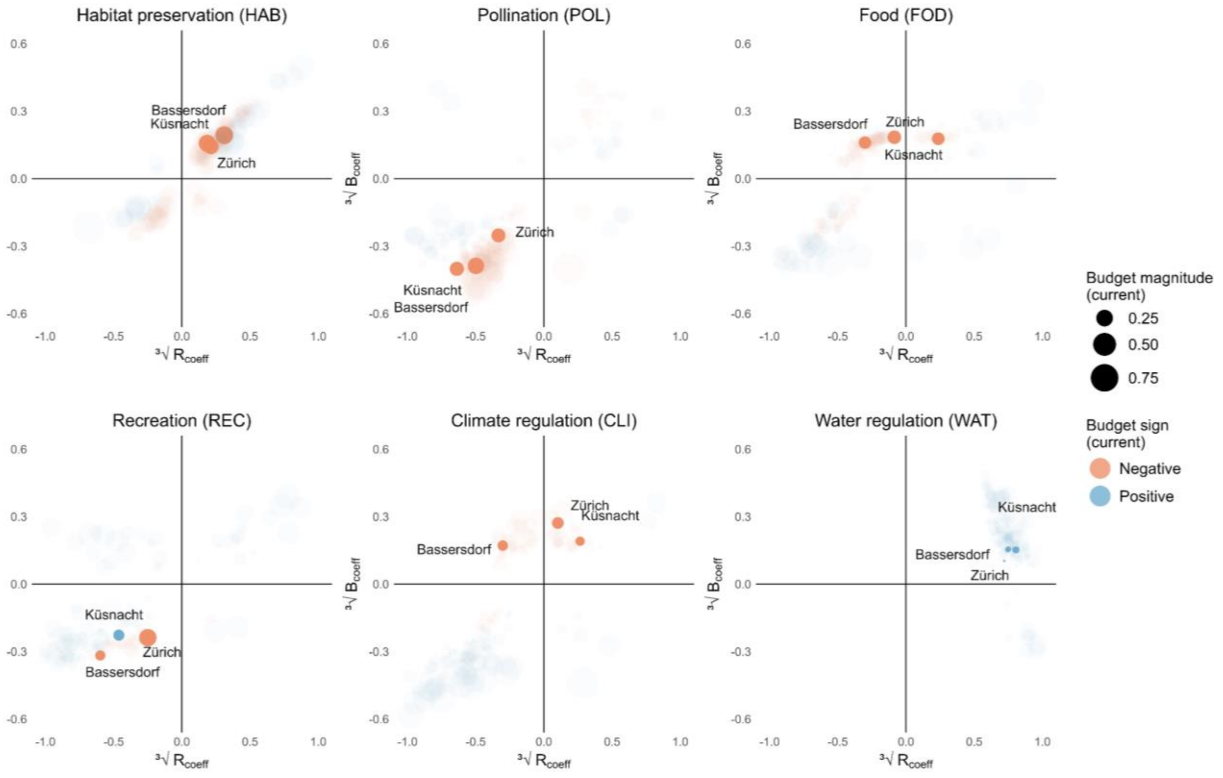

Figure F1: Temporal trends in NCPs budget and ratio for the three municipalities of the Urban-Fields Nexus cluster: Zurich, Küsnacht and Bassersdorf. The dots of all the other municipalities are semi-transparent. For a detailed description see Figure 5.

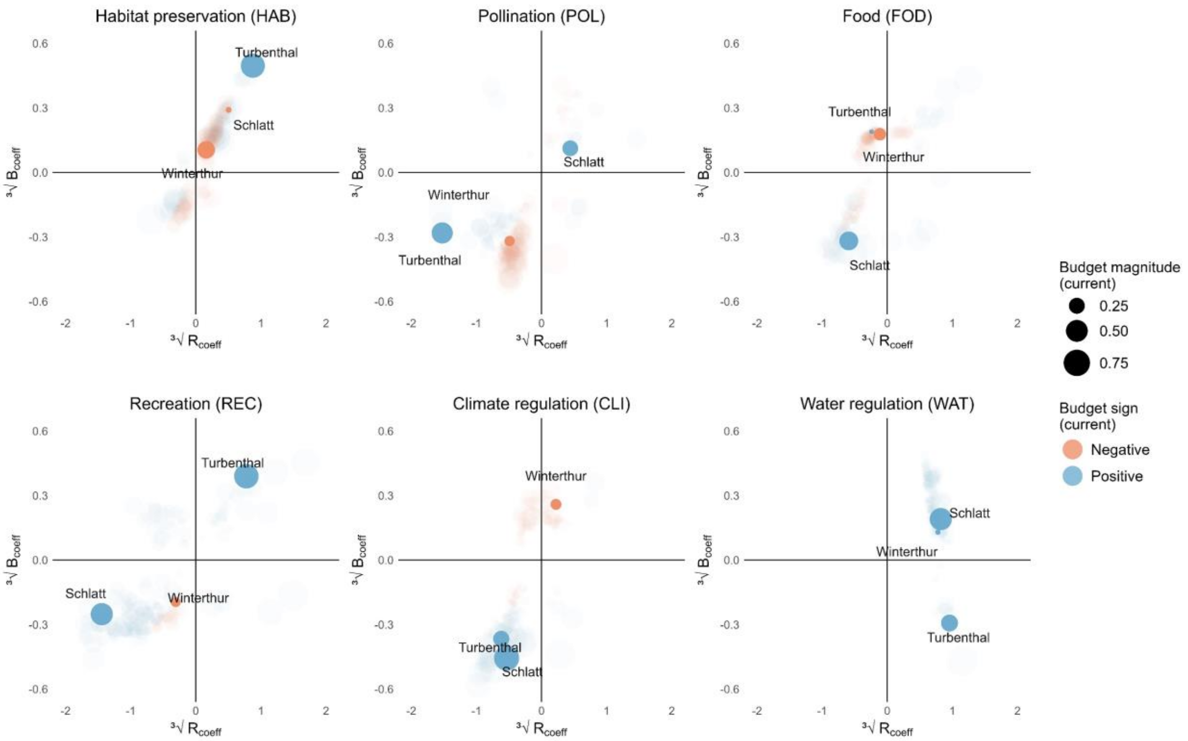

Figure F2: Temporal trends in NCPs budget and ratio for the three municipalities of different clusters: Winterthur (Urban-Fields Nexus), Schlatt (Agrofood Patches) and Turbenthal (Abundance Highlands). The dots of all the other municipalities are semi-transparent. For a detailed description see Figure 5.
